# Metabolomics of Human Amniotic Fluid and Maternal Plasma during Normal Pregnancy

**DOI:** 10.1371/journal.pone.0152740

**Published:** 2016-04-12

**Authors:** Magdalena Orczyk-Pawilowicz, Ewa Jawien, Stanislaw Deja, Lidia Hirnle, Adam Zabek, Piotr Mlynarz

**Affiliations:** 1 Department of Chemistry and Immunochemistry, Wroclaw Medical University, Wroclaw, Poland; 2 Department of Chemistry, Wroclaw University of Technology, Wroclaw, Poland; 3 1^st^ Department and Clinic of Gynaecology and Obstetrics, Wroclaw Medical University, Wrocław, Poland; 4 Faculty of Chemistry, Opole University, Opole, Poland; National Research Council of Italy, ITALY

## Abstract

Metabolic profiles of amniotic fluid and maternal blood are sources of valuable information about fetus development and can be potentially useful in diagnosis of pregnancy disorders. In this study, we applied ^1^H NMR-based metabolic profiling to track metabolic changes occurring in amniotic fluid (AF) and plasma (PL) of healthy mothers over the course of pregnancy. AF and PL samples were collected in the 2^nd^ (T2) and 3^rd^ (T3) trimester, prolonged pregnancy (PP) until time of delivery (TD). A multivariate data analysis of both biofluids reviled a metabolic switch-like transition between 2^nd^ and 3^rd^ trimester, which was followed by metabolic stabilization throughout the rest of pregnancy probably reflecting the stabilization of fetal maturation and development. The differences were further tested using univariate statistics at α = 0.001. In plasma the progression from T2 to T3 was related to increasing levels of glycerol, choline and ketone bodies (3-hydroxybutyrate and acetoacetate) while pyruvate concentration was significantly decreased. In amniotic fluid, T2 to T3 transition was associated with decreasing levels of glucose, carnitine, amino acids (valine, leucine, isoleucine, alanine, methionine, tyrosine, and phenylalanine) and increasing levels of creatinine, succinate, pyruvate, choline, *N*,*N*-dimethylglycine and urocanate. Lactate to pyruvate ratio was decreased in AF and conversely increased in PL. The results of our study, show that metabolomics profiling can be used to better understand physiological changes of the complex interdependencies of the mother, the placenta and the fetus during pregnancy. In the future, these results might be a useful reference point for analysis of complicated pregnancies.

## Introduction

Pregnancy is associated with the onset of many adaptation processes that are likely to change over the course of gestation [1]. In particular, metabolic composition of blood and amniotic fluid should reflect these biochemical dynamics. Maternal blood remains in constant exchange with fetus through the placenta providing nutrients required for growth and development. Amniotic fluid originates from maternal, fetal and placental tissues therefore its metabolic profile is the net result of metabolite synthesis/degradation, fetal maturation (particularly of the kidneys and lungs), and biochemical exchanges [2,3,4]. Because of their biochemical nature plasma and amniotic fluid should recapitulate the physiological processes of fetal development what makes them an extremely valuable material for fetal health diagnostics [5,6].

NMR-based metabolomics is an established technique for studying complex biological samples (e.g., plasma, urine or amniotic fluid) [7–11]. Metabolomics exploits high-throughput analytical measurements to identify and quantify metabolites allowing to describe dynamic changes in phenotype and system homeostasis [12]. The metabolic profiles of amniotic fluid and the maternal plasma offer new insights to better understand the organ systems and biofunctions that contribute to fetal well-being during normal pregnancy [6,9,13,14]. The great advantage of such an approach is that all of the metabolites (being present in high enough concentration in the biological sample) are measured simultaneously, and a pattern of several metabolites (metabolic profile) can be more informative than the measurement of a single metabolite/analyte [15]. It should be however emphasized that the use of metabolomics in prenatal and perinatal medicine is still in its infancy [9].

Current understanding of the relationship between metabolite composition of amniotic fluid and maternal plasma during normal pregnancy is still not complete. Few studies have monitored healthy pregnancy using either amniotic fluid or plasma but these biofluids have not been studied in combination by metabolomics [16–19]. Other reports have mainly focused on selected stages or disorders of pregnancy [20–22], such as fetal malformations [23], gestational diabetes mellitus [24], macrosomia [25], preeclampsia [26,27], preterm delivery [13,14], spina bifida [28] and very-low birth weight [29], and did not clearly show the changes that occur between trimesters. To the best of our knowledge, the present study is the first combined metabolomics analysis of amniotic fluid and maternal plasma during the progression of normal pregnancy, starting at the 2^nd^ trimester (T2) and continuing through the 3^rd^ trimester (T3), prolonged pregnancy (PP) until time of delivery (TD). The aim of our investigation was to characterize the metabolic signature of human amniotic fluid and maternal plasma over the course of normal pregnancy. This was achieved by analyzing amniotic fluid and maternal plasma using ^1^H NMR-based metabolomics.

In the study reported in this manuscript the biggest metabolic differences in both amniotic fluid and maternal plasma were detected between 2^nd^ and 3^rd^ trimester suggesting the metabolic switch-like transition occurring in fetus and mother organism.

## Materials and Methods

### Patients and methods

This study was conducted at the Department of Obstetrics and Gynaecology, Wrocław Medical University, Wrocław, Poland. All of the samples were collected after written informed consent was obtained from the individual women. The study was approved by the Ethics Committee at Wrocław Medical University, Wrocław, Poland (protocol number KB-227/2011). Amniotic fluid (AF) was collected by transabdominal amniocentesis under ultrasonographic guidance during the 2^nd^ (T2) and 3^rd^ trimesters (T3), and prolonged pregnancy (PP), and using transvaginal amniotomy during delivery (TD).

The samples used for the current study consisted of the fluids (AF- 73 samples and PL -63 samples) that remained after the performance of the routine diagnostic procedures performed for a) genetic abnormalities in previous pregnancies, b) family history, c) maternal age <35 years. All samples used for this study were obtained from cases that proved negative at diagnostic examination.

In all cases, an accurate gestational age was established by the last menstrual period confirmed by ultrasonographic evaluation. There were no signs of inflammation, vaginal infection or chorioamnionitis. Immediately after amniocentesis, all of the amniotic fluid samples were centrifuged at 3000xg for 20 minutes to separate the cells from the supernatant, which was then aliquoted and stored at −76°C until use. Amniotic fluid samples that were contaminated with blood or meconium were discarded. All of the participating women were healthy (age range 21–38 y.o.) and delivered healthy newborns without malformations, chromosomal abnormalities or postmaturity syndrome.

All of the blood samples (PL) were collected into anticoagulant-treated tubes (sodium citrate) in the morning 12 hours after the last meal and were gently mixed to dissolve the anticoagulant. Next, blood samples were centrifuged at 2000xg for 15 minutes to obtain the plasma, which was then aliquoted and stored at -76°C until use.

### Sample preparation

Prior to the analysis, aliquots of human amniotic fluid and plasma were thawed at room temperature for 1 hour. For the NMR measurements, 200 μL of plasma was mixed with 400 μL of saline solution (NaCl 0.9% in 10% D_2_O). The mixture was centrifuged at 10,000xg for 10 minutes, and then 550 μL of supernatant was transferred into a 5-mm NMR tube. For the amniotic fluid analysis, 200 μL of 0.5 M sodium phosphate buffer (pH = 7 containing 33.3% of D_2_O, 0.5 mM 3-(trimethylsilyl)-2,2’,3,3’-tetradeuteropropionate sodium salt TSP-d4 (TSP) as an internal standard and 3 mM sodium azide to prevent microbial contamination) was added to 400 μL of AF. The mixture was then centrifuged at 10,000xg for 10 minutes, and 550 μL of the supernatant was transferred into a 5-mm NMR tube.

### ^1^H NMR measurements

The NMR spectra were recorded using Bruker Avance spectrometer operating at the proton frequency of 600.58 MHz. Sample temperature was set at 300 K. A one-dimensional Carr-Purcell-Meiboom-Gill (CPMG) NMR spin echo pulse sequence with water suppression was employed to filter out broad spectral resonances arising from the macromolecules.

For each AF sample, 256 following scans were collected using 3.5 s relaxation delay and an acquisition time of 1.36 s, resulting in 32k data points and a spectral width of 20.01 ppm. The spectra were processed with a line broadening of 1.0 Hz and were manually phased and baseline-corrected using the Topspin 1.3 software (Bruker, GmBH, Germany). The spectra were referenced to the TSP signal (δ = 0.00 ppm), and alignment was performed using the icoshift algorithm [30]. Finally, after the residual water (4.60–5.00 ppm), urea and α-proton of glucose (5.00–6.00 ppm) regions were removed, the dataset was binned into 7951 integrals of equal width (0.001 ppm).

For each plasma sample, 128 following scans were collected using 3.5 s relaxation delay and acquisition time of 2.73 s, resulting in 32k data points and a spectral width of 20.01 ppm. The spectra were processed with a line broadening of 0.3 Hz and were manually phased and baseline-corrected using the Topspin 1.3 software (Bruker, GmBH, Germany). The whole spectrum was referenced to the α-glucose signal (δ = 5.225 ppm) and alignment was performed using the icoshift algorithm [30]. Finally, after the residual water region (4.40–5.00 ppm) and citrate (2.48–2.69 ppm) were selectively removed, the dataset was binned into 8716 integrals of equal width (0.001 ppm).

### Multivariate data analysis and statistical analysis

All of the spectra were normalized using the probabilistic quotient normalization (PQN) method and the Matlab program [31]. The data matrices containing either the binned spectra or the areas of the metabolites were transferred into SIMCA-P (v 13.0, Umetrics, Sweden), where the principal component analysis (PCA) was conducted. The data were scaled using Pareto scaling (binned spectra) or unit variance scaling (quantified metabolites). In the case of quantified metabolites (signal areas) only the signals without overlap were used. Orthogonal Projections to Latent Structures-Discriminant Analysis (OPLS-DA) was used for testing the differences between metabolic profiles and considered statistically significant at α = 0.001 (CV-ANOVA). STATISTICA software (v 10, StatSoft, Tulsa, USA) was utilized for the statistical analysis of the quantified metabolites. In a univariate analysis, statistical importance was determined using Student's *t*-test because the data were characterized by normal distribution (S1 Matrix; S2 Matrix). The signal originating from the β-proton of glucose was removed during the multivariate analysis but considered in univariate statistics. Bonferroni’s correction for multiple testing was used in the statistical analysis thus, a variable was considered statistically significant at α = 0.001.

## Results

**Fig 1:**
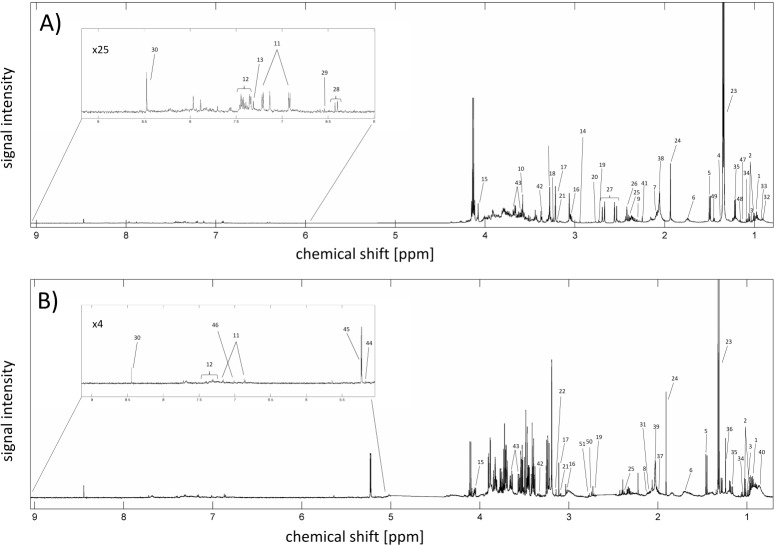
**A representative example of the** ^**1**^**H NMR spectrum of amniotic fluid (A) and plasma (B).** The numbers in the figure correspond to the numbers in Table A in S1 File. 1 –leucine, 2 –valine, 3 –isoleucine, 4 –threonine, 5 –alanine, 6 –lysine, 7 –methionine, 8 –glutamine, 9 –glutamate, 10 –glycine, 11 –tyrosine, 12 –phenylalanine, 13 –histidine, 14 –N,N—dimethylglycine, 15 –creatinine, 16 –creatine, 17 –choline, 18 –carnitine, 19 –dimethylamine, 20 –sarcosine, 21 –dimethylsulfone, 22 –trimethylamine N-oxide (TMAO), 23 –lactate, 24 –acetate, 25 –pyruvate, 26 –succinate, 27 –citrate, 28 –urocanate, 29 –fumarate, 30 –formate, 31 –acetoacetate, 32–2-hydroxyisovalerate, 33–2-hydroxybutyrate, 34 –isobutyrate, 35–3-hydroxybutyrate, 36–3-hydroxyisovalerate, 37 –proline, 38 –N-acetyl groups, 39 –NAC, 40 –VLDL/LDL, 41 –acetone, 42 –methanol, 43 –glycerol, 44 –mannose, 45 –glucose, 46 –AU1, 47 –AU2, 48 –PU1, 49 –PU2.

Table 1 reports the number of PL, AF and paired samples (AF and PL collected from the same mother) obtained at different epochs of pregnancy and analyzed by ^1^H NMR spectroscopy. Representative examples of the ^1^H NMR spectra of amniotic fluid and plasma are shown in Fig 1A and 1B. All of the spectra included several metabolite groups, such as amino acids, lipids, organic acids, carbohydrates and nucleotides. A total of 34 metabolites in the amniotic fluid and 30 metabolites in the plasma were identified (Table A in S1 File). Additionally, two compounds in each biofluid remained unassigned. For further analysis, the unassigned metabolites in the AF were marked as AU1, AU2, while those in the PL were marked as PU1 and PU2.

**Table 1:** The characterization of the amniotic fluid and plasma samples.

Sampling stages	Mean gestational age at sampling in weeks	PL	AF	Paired samples*
T2	15.4 ±0.96	7	6	1
T3	37.7±1.68	16	21	15
TD	40.1±1.13	29	33	26
PP	41.3±0.46	11	13	8

*PL and AF samples obtained from the same patient

The resonances of metabolites were identified using assignments published in the literature [28,32,33] and the on-line databases (www.hmdb.ca) [34]. Generally, inspection of ^1^H NMR spectra of AF and PL divided into the chemical shift regions showed the greatest abundance of the following metabolites: aliphatic region (0–3 ppm), the amino acids (leucine, isoleucine, valine, alanine, threonine (AF), lysine, glutamate (AF), proline, glutamine and methionine (AF)) and the organic acids (2-hydroxybutyrate (AF)), lactate, acetate, pyruvate, succinate (AF) acetoacetate (PL) and citrate (AF)). In all of the analyzed samples, the carbohydrate profiles (3–5.5 ppm) were composed mainly of glucose with an additional small fraction of mannose (PL). In the aromatic region (5.5–10 ppm), the aromatic amino acids (tyrosine, phenylalanine and histidine (AF)) and organic acids (fumarate (AF), formate) were the most abundant. Moreover, all of the amniotic fluid spectra showed the presence of urocanate [6], which was confirmed using Statistical Total Correlation Spectroscopy (STOCSY). Briefly, the resonances putatively assigned to urocanate molecule exhibited high correlation coefficient values in analyzed set of spectra (Fig A in S1 File).

**Fig 2:**
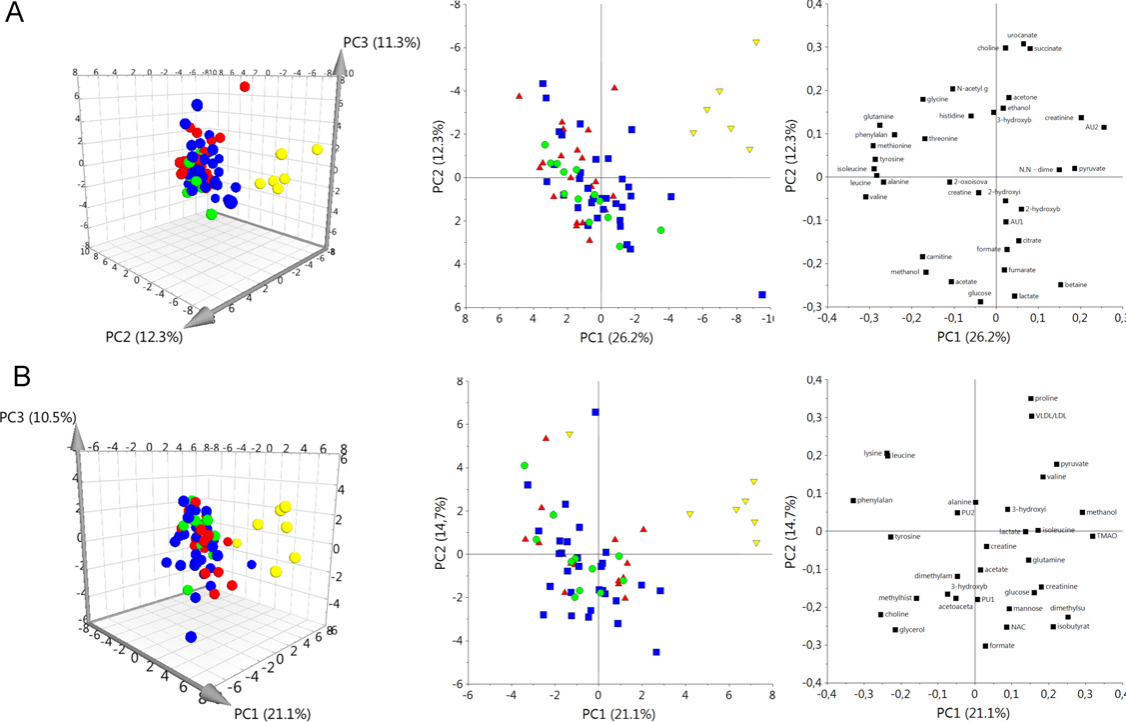
**The PCA results obtained using quantified metabolites (signal areas) for amniotic fluid (A) and plasma (B).** Pregnancy stages. Yellow inverted triangles– 2^nd^ trimester (T2), red triangles– 3^rd^ trimester (T3), blue squares–delivery (TD) and green circles–prolonged pregnancy (PP).

In order to visualize the data a chemometric approach was used. Since plasma and amniotic fluid samples were not paired the unsupervised multivariate data analysis was carried out separately for amniotic fluid and plasma data sets. In both cases PCA was conducted based only on the metabolite signals that were quantified and additionally based on the whole binned spectra (Fig B in S1 File). Score and loadings plots obtained for both biofluids are shown in Fig 2.

Regardless of analyzed biofluid the observed grouping was similar, with well separated T2 group and high degree of overlap between rest of the samples (T3, TD and PP groups). This was fully achieved by first two principal components (PC). Introduction of additional PCs did not resolve any novel grouping patterns (Fig 2). The analysis of AF loadings plot showed that all of the quantified amino acids were grouped close together and thus exhibit similar pattern (Fig 2A**)**. Conversely, such trend was not observed in PL (Fig 2B).

To further investigate whether metabolic profiles of amniotic fluid and plasma are changing between T2, T3, TD and PP periods a discriminant analysis was employed. The following set of comparisons was subjected to OPLS-DA modeling: T3 vs. T2; TD vs. T3; PP vs. T3; and PP vs. TD. Consistent with PCA results, a clear separation between T2 and all other groups was detected. All three discriminant models involving T2 group (T2 vs T3, T2 vs TD, T2 vs PP) exhibited high level of statistical significance and high values of R2X(cum) and Q2Y(cum) parameters (Table B in S1 File) proving that T2 period is characterized by unique AF and PL metabolic profile. Similar, trend was observed when discriminant analysis was carried out using whole profile (binned spectra) (Table C in S1 File). Conversely, all other comparisons were not-statistically significant (Table B in S1 File) suggesting limited metabolic changes in composition of either AF or PL during transition from T3 to PP period. Again, analogous tendency was observed when whole profile was used for chemometric modeling (Table C in S1 File).

For determination of statistical significance of individual metabolites a univariate statistical analysis was performed with Bonferroni’s correction for multiple testing. Metabolites that were statistically significant in at least one comparison were listed in Table 2. The highest number of significantly affected metabolites was observed in the T3 vs. T2 comparison, in which sixteen metabolites in the AF (leucine, valine, isoleucine, alanine, methionine, tyrosine, phenylalanine, *N*,*N*-dimethylglycine, carnitine, creatinine, succinate, pyruvate, urocanate, glucose, choline and AU2) and five metabolites in the PL (pyruvate, 3-hydroxybutyrate, acetoacetate, glycerol, and choline) were found.

**Table 2:** Differences of metabolites (expressed as percentage) between the four gestational age periods.

Metabolites	Biofluid	T3 vs. T2	TD vs. T3	PP vs. T3	PP vs. TD
Leucine	AF	**-52.81**	29.34	7.17	-17.14
Valine	AF	**-62.29**	24.84	2.54	-17.86
Isoleucine	AF	**-50.68**	25.87	14.17	-9.29
Alanine	AF	**-44.60**	1.92	-4.21	-6.02
Methionine	AF	**-44.41**	11.91	1.02	-9.73
Tyrosine	AF	**-46.70**	17.81	6.78	-9.36
Phenylalanine	AF	**-32.40**	15.78	9.37	-5.54
*N*,*N*-dimethylglycine	AF	**55.26**	-1.89	-3.54	-1.68
Carnitine	AF	**-44.37**	-2.00	-17.65	**-15.97**
Creatinine	AF	**84.92**	-5.84	1.99	8.32
Citrate	AF	1.69	7.23	**-26.18**	-31.16
Succinate	AF	**114.06**	-0.57	-11.19	-10.68
Pyruvate*	AF	**238.48**	-22.64	**-36.35**	-17.74
Pyruvate*	PL	**-45.37**	13.27	5.34	-6.99
Acetoacetate	PL	**61.32**	2.88	16.52	14.04
Urocanate	AF	**672.58**	7.19	15.23	7.50
3-hydroxybutyrate	PL	**101.48**	22.84	-15.85	-31.50
Glycerol	PL	**49.83**	1.48	0.95	0.52
Glucose	AF	**-50.24**	24.23	-13.42	-30.31
Choline*	AF	**32.71**	0.84	11.23	10.31
Choline*	PL	**176.58**	7.34	6.11	-1.15
AU 2	AF	**304.66**	-8.97	-9.95	-1.08
Lac/Pyr	AF	**-72.34**	20.38	42.56	18.42

The percent difference was calculated using the mean values of the relative signal integrals in each group. Bold type indicates a statistical significance of p<0.001 according to Student’s *t*-test.

* Metabolites present in both biofluids.

Changes in the other comparisons were much less pronounced and limited to AF only. Two metabolites namely (citrate and pyruvate) were significantly down regulated in PP vs T3 comparison, while in PP vs TD carnitine was decreased. No statistically significant changes were detected in TD vs T3 comparison.

**Fig 3:**
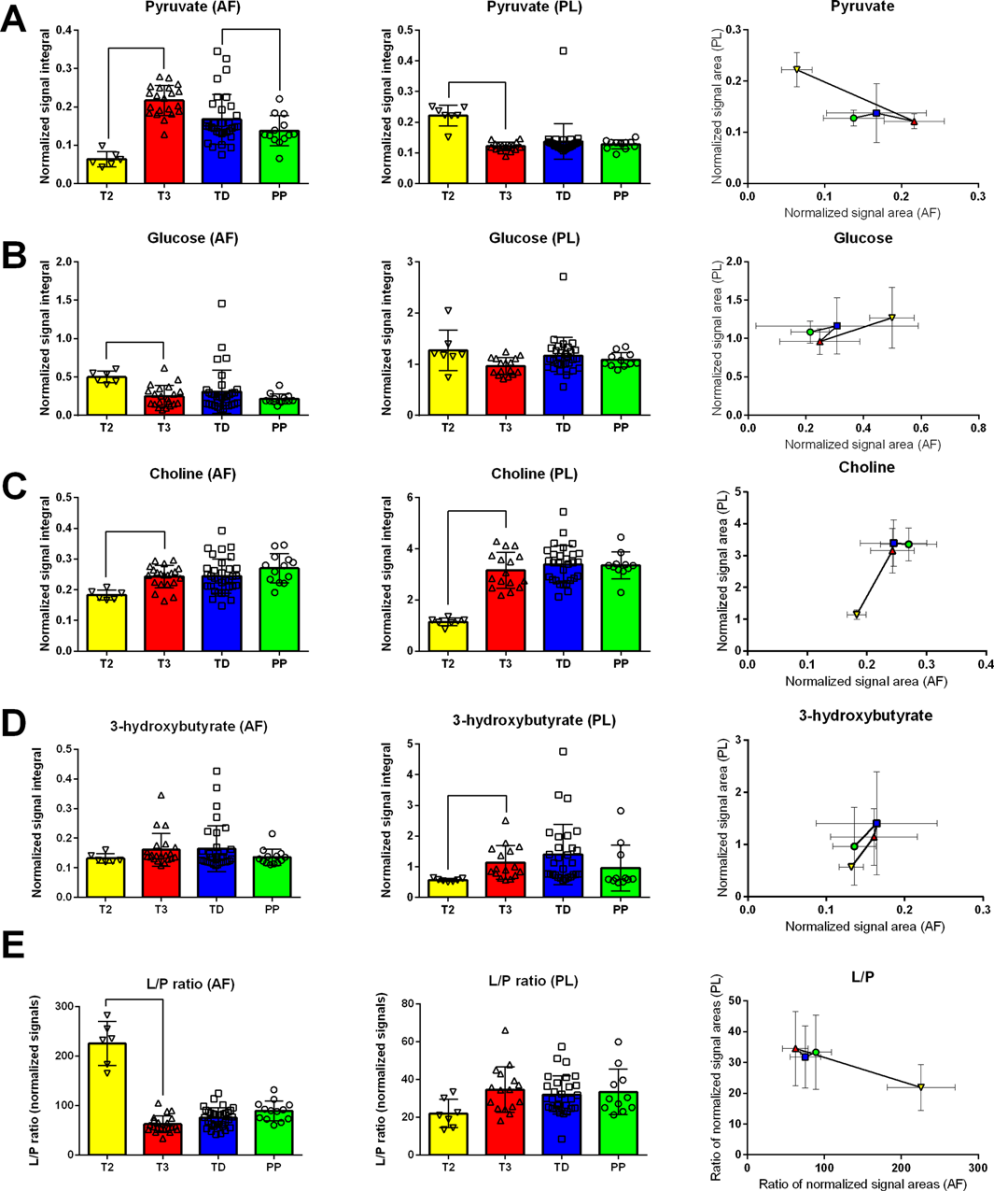
**Significantly changed metabolites that were detected in both biofluids: pyruvate (A), glucose (B), choline (C), 3-hydroxybutyrate (D) and lactate to pyruvate ratio (E).** Bar graphs obtained for amniotic fluid (left) and for plasma (middle). Relationship plot (right)—symbol denotes to average and error bars denotes to standard deviation of the normalized signal values for each group. T2 –second trimester, T3 –third trimester, TD–time of delivery, PP–prolonged pregnancy.

It was not possible to perform a correlation analysis due to the fact that most of plasma and amniotic fluid samples were not paired. However, for metabolites that were detected in both biofluids (and significantly changed in at least one comparison) a relationship analysis was conducted by plotting group average and standard deviation values (Fig 3). For pyruvate and choline a strong relationship between changes in plasma and amniotic fluid was observed. While pyruvate was increasing in AF it was decreasing in PL during T2 to T3 transition. This change was than fallowed by stabilization in PL pyruvate level and moderate decrease in AF (from T3 to PP). In case of choline a simultaneous significant increase was detected in both AF and PL during T2 to T3 transition. No strong intra-biofluid relationship was found neither for 3-hydroxybutyrate nor for glucose.

Due to availability of lactate and pyruvate in PL and AF, a lactate to pyruvate ratio (L/P) was calculated. The L/P ratio is expressed in arbitrary units since absolute concentrations were not available. Metabolite quantification was conducted using only signals without overlap thus, calculated L/P ratio values can be used only for relative comparisons and do not reflect physiological level of this parameter. Driven mostly by changes in pyruvate the L/P ratio was significantly diminished in AF and noticeably elevated (although not statistically significant, p value = 0.0066 at α = 0.001) in PL in T2 vs T3 comparison. L/P ratio was stable in T3, TD and PP groups in both biofluids. The 3-hydroxybutyrate/acetoacetate (3-HB/AcAc) ratio was calculated only for PL samples, but no differences were detected (data not shown).

## Discussion

Collection of AF by amniotomy has certain limitations that can influence the composition of amniotic fluid metabolic profile. In particular the risk of introducing substances from vaginal environment should be taken into consideration. Our analysis did not show statistically significant differences between T3 and TD samples. which were collected using transabdominal amniocentesis or amniotomy respectively. Therefore, it is rather unlikely that contaminating metabolites were a substantial constituent of AF metabolic profile measured using ^1^H NMR spectroscopy. However, in case of a more sensitive analytical platform e.g. LC-MS such contamination likely will be detected.

The strongest differences in the metabolic composition of both analyzed biofluids were found between the T2 and T3 stages of normal pregnancy (Table 2, Fig 2), while with the progression of pregnancy, metabolic stabilization becomes evident. This interesting finding suggests a metabolic switch-like transition occurring in mother’s system between T2 and T3 periods. Therefore, our observations are most likely related to the change in fetus growth dynamics, namely transition into fast weight-gain phase, which requires considerably higher rates of anabolic processes. In contrast to plasma, the amniotic fluid showed significant decrease in the levels of amino acids during transition mentioned above (Table 2, Fig B in S1 File). This is likely associated with fetal maturation and the increased demand for elementary building blocks, which are necessary for protein synthesis [35] and might be utilized for many other processes required to maintain fetal homeostasis during rapid growth [36]. Amino acids are crucial for proper energy balance and maintenance of TCA cycle span by providing carbon backbone exchange *via* various anaplerotic and cataplerotic pathways [37]. It is known that a shortage of amino acids, may strongly influence fetal protein biosynthesis [36].

It is established that during first two-thirds of gestation the mother’s organism is in anabolic condition, while during the T3 maternal metabolism is switched towards catabolic activity [38], which is than opposite from the intensive anabolic processes occurring in fetus.

An example of catabolic/anabolic switch is the decrease in the level of glucose in amniotic fluid, which overlaps with increasing energy demand during the pregnancy progression. It is known that mothers can become hypoglycemic even though their gluconeogenesis rates are increased [39]. This is due to fetus high glucose uptake through the placenta. Therefore, small decrease in plasma glucose levels in T3 might be attributed with depleting glycogen stores, though influence of diet cannot be excluded.

The significantly increased mother’s plasma glycerol in T3 vs T2 comparison is most likely a result of triglycerides breakdown caused by high lipolitic activity of adipose tissue [38]. Glycerol can be further utilized either as a substrate for gluconeogenesis and triglycerides biosynthesis [38] or to help to maintain proper redox balance being a part of glycerol phosphate shuttle [40]. Interestingly it was suggested that glycerol can be a preferential substrate for gluconeogenesis during pregnancy [41] however, since mentioned study compared glycerol with pyruvate and alanine, it was more an evaluation of pyruvate carboxylase anaplerotic flux. Triglycerides hydrolysis results in a high concentration of free fatty acids that undergo beta oxidation which is supplementing TCA cycle and ketone bodies synthesis by providing acetyl-CoA moieties. Indeed, we observed significantly elevated levels of 3-hydroxybutyrate and acetoacetate in T3 vs T2 comparison [42]. In contrast, carnitine, which is required for activation of fatty acids and relegating them towards beta oxidation in mitochondria was found to be consequently decreasing throughout the course of pregnancy (Table 2). Moreover, the sufficient level of carnitine was postulated to have an important role in fetal growth maturation by increased birth weight and postnatal growth rate [43].

TCA cycle is a nexus point of central carbon metabolism elegantly balancing amino acids, carbohydrates and lipids metabolism [44]. Besides free fatty acids, pyruvate is one of the major sources of acetyl-CoA for TCA cycle. Pyruvate fluctuation in plasma moderately corresponded to the level of glucose, which was higher in T2 than in the subsequent periods of pregnancy. Surprisingly, the pyruvate level in the amniotic fluid exhibited a reverse trend against glucose (Fig 3A and 3B).

All discussed pathways: beta-oxidation, ketone bodies production, glycolysis and TCA cycle are linked *via* oxidative phosphorylation to cellular redox state [45]. *In vivo* redox readout can be challenging, yet one of the most common approaches is utilization of redox pairs. In particular lactate/pyruvate (L/P) and 3-hydroxybutyrate/acetoacetate (3-HB/AcAc) ratios are proven to be valuable. L/P is considered to reflects cytosolic, while 3-HB/AcAc mitochondrial redox potential. There is an inverse relationship between L/P and oxygen consumption [46]. Albeit not being statistically significant after Bonferroni’s correction for multiple testing (p value = 0.0066 at α = 0.001), the mothers plasma L/P ratio was noticeably elevated between T2 and T3 and stayed unchanged in TD and PP. It has been reported that pregnant women have decreased levels of pyruvate in blood comparing to non pregnant control resulting in elevated L/P ratio [47]. Moreover, L/P is even further elevated in twin comparing to singleton pregnancies [48] due to anaerobic metabolism. Interestingly, opposite to plasma, amniotic fluid L/P ratio was significantly (p = 0.0002) decreased between T2 and T3. Decrease in L/P ratio is considered to correspond to the elevated oxygen consumption thus, our observations might reflect the increase in fetus aerobic metabolism. In conclusion, reverse L/P trends in AF and PL suggest that transition from 2^nd^ to 3^rd^ trimester is coupled with the change in oxygen utilization by fetus and mother body.

Changes in choline concentration were evident in both the AF and the PL. Increased choline level might be correlated with higher phospholipid demand for the fetus brain development during later periods of pregnancy [49]. The same trend, but for amniotic fluid only, was found for *N*,*N*-dimethylglycine, which is an intermediate metabolite in glycine’s biosynthesis from choline.

Creatinine, a marker of the function and maturation of fetal kidneys [50], increased significantly during T3 and then remain stable once the kidneys have fully matured. Note that the composition of AF reflects the fetal renal system, thus, the degree of fetal renal maturation should significantly influence the level of almost all of the metabolites in the amniotic fluid [50,2,51].

To the best of our knowledge, this is for the first time that urocanate has been observed in amniotic fluid using ^1^H NMR. We confirmed presence of urocanate by STOCSY analysis [6, 52]. This metabolite is an important photoprotectant predominantly located in the skin. It is generated from histidine by enzyme histidinase and contributes to acidic mantle [53]. Throughout most of pregnancy fetus skin is transparent thus high levels of urocanate in AF in T2 might reflect rapid growth of skin and requirement for photoprotectants.

It is worth noticing that even though amniotic fluid samples were not paired with plasma samples a high degree of agreement in metabolic trends was observed. This was especially true in case of transition from T2 to T3 period (Fig 3). Correlation between plasma and amniotic fluid metabolites opens possibilities to investigate fetus metabolic status by mother's plasma metabolomics monitoring. It is highly probable that more metabolic trends could be identified by using paired samples. Yet, for medical and ethical reasons, it is extremely difficult to obtain amniotic fluid from healthy mothers.

Even though in our study the biggest differences were observed between T2 and T3, we cannot exclude the possibility that even more pronounced transition occurs between T1 and T2. A substantial decrease in lysophosphatidylcholines and lysophosphatidylethanolamines plasma content was found between T1 and T2 [18]. Furthermore, a number of amino acids, non-esterified fatty acids and acylcarnitines was shown to change between 1^st^ and 2^nd^ trimester of healthy pregnancy [19].

## Conclusions

The second trimester was easily distinguished from the other investigated periods while no significant discrimination could be achieved between other groups. A metabolic switch-like transition in both biofluids was discovered between second and third trimester, which was followed by metabolic stabilization throughout the rest of pregnancy. To the best of our knowledge, this study provides the first metabolomics analysis of amniotic fluids and plasma collected from healthy pregnant women at different stages of pregnancy and during delivery. In the future, these results might be a useful reference point for analysis of complicated pregnancy and potentially transfer to some extent to the clinical arena.

## Supporting Information

S1 File All figures and tables of Supporting Information. Legend: Fig A. STOCSY analysis of the amniotic fluid exhibited high correlations between the urocanate signals. Fig B. The PCA score plot results obtained for the ^1^H NMR data corresponding to the pregnancy stages. Green circles–prolonged pregnancy (PP), blue squares–delivery (TD), red triangles– 3^rd^ trimester (T3) and yellow inverted triangles– 2^nd^ trimester (T2). Table A. Metabolites identified in amniotic fluid and plasma using ^1^H NMR spectroscopy. Table B. Parameters of OPLS-DA model (two components) calculated using quantified metabolites (signal areas). Table C. Parameters of OPLS-DA model (two components) calculated using binned spectra.
(RAR)

S1 Matrix The data matrix of AF, which contains all necessary data needed to replicate experiment. (XLSX)

S2 Matrix The data matrix of PL, which contains all necessary data needed to replicate experiment. (XLSX)
